# Epidemiological Evaluation of Notifications of Environmental Events in the State of São Paulo, Brazil

**DOI:** 10.3390/ijerph110707508

**Published:** 2014-07-21

**Authors:** Telma de Cassia dos Santos Nery, Rogerio Araujo Christensen, Farida Pereira, Andre Pereira Leite

**Affiliations:** Division of Diseases Caused by Environment, Epidemiological Surveillance Center, State Department of Health, Av. Dr. Arnaldo 351, Sao Paulo 01246-000, Brazil; E-Mails: rchristensen54@gmail.com (R.A.C.); faridacon@gmail.com (F.P.); andrepleite@gmail.com (A.P.L.)

**Keywords:** epidemiological surveillance, environmental health, notification of chemical substances, epidemiological investigation, environmental factors

## Abstract

Increasing urbanization across the globe, combined with an increased use of chemicals in various regions, contributes to several environmental events that influence environmental health. Measures that identify environmental factors and events should be introduced to facilitate epidemiological investigations by health services. The Brazilian Ministry of Health published a new list of notifiable diseases on 25 January 2011 and introduced environmental events as a new category of notifiable occurrences. The Center for Epidemiologic Surveillance in State of Sao Paulo, Brazil, created an online notification system that highlights “environmental events”, such as exposure to chemical contaminants, drinking water with contaminants outside of the recommended range, contaminated air, and natural or anthropogenic disasters. This paper analyzed 300 notifications received between May 2011 and May 2012. It reports the number of notifications with event classifications and analyzes the events relating to accidents with chemical substances. This paper describes the characteristics of the accidents that involved chemical substances, methods used, types of substances, exposed population, and measures adopted. The online notification of environmental events increases the analysis of the main events associated with diseases related to environmental chemicals; thus, it facilitates the adoption of public policies to prevent environmental health problems.

## 1. Introduction

Increasing urbanization across the globe, combined with increased use and movement of chemicals in several regions by various means of transport, contributes to environmental events that influence environmental health [1]. Changes in current industrial processes throughout the world are determining factors in several environmental areas, including soil, air, and water [2]. Across the planet, many areas have been identified as contaminated. The issues that surround environmental contamination by chemicals and hazardous waste, specifically in the case of soil, are associated with a model of industrial development [3]. Thousands of contaminated areas have been identified in industrialized countries:

- Germany: about 300,000
- United States: about 500,000
- Austria: about 21,000
- Denmark: about 10,700
- Switzerland: about 4000

In Brazil, the Ministry of Health has estimated that two million people have potentially been exposed. Measures to identify environmental factors and events should be introduced, facilitating epidemiological investigations by health services [4,5]. The Ministry of Health of Brazil published an update list of notifiable diseases on 25 January 2011 and introduced the notification of environmental events.

The Center for Epidemiologic Surveillance in the State of Sao Paulo created an online notification system which highlights “environmental events”, such as the exposure to chemical contaminants, drinking water with contaminants outside of the recommended range, contaminated air, and natural or anthropogenic disasters [6].

The State of Sao Paulo, Brazil has 645 counties and 42 million people. It represents 33% of the gross national product (GNP) and is the largest industrial region, about 120,000 industries, 50% of the industries in the country, which includes a group of 1900 industries that have accounted for 90% of the most serious and dangerous industrial pollution.

Other related informations about the State of Sao Paulo:

- 10,000 retail fuel stations and systems;
- 4000 km of pipelines (*i.e.*, gas and oil);
- 33,000 kilometers of paved roads;
- Four oil refineries;
- Two major seaports of Brazil;
- The industrial park of São Paulo, produces over 50,000 tons of industrial solid waste per day;
- 22 million vehicles in the fleet of the State;
- 4500 identified contaminated areas (2012);
- Annual average of more than 450 accidents involving chemical substances, 80% of them by road system.

The Epidemiological Surveillance Center within the Division of Diseases Occasioned by Environment—DOMA has a team of nine individuals composed of multiple experienced professionals, including doctors, civil engineers and biologists.

### 1.1. Current Priorities of the Division

- Increase the prominence of the topic of environmental health by identifying epidemiological data that requires interventions, attending events, and publicizing the issue;
- Empower the Health Services from Unified Health System (SUS) network to work on environmental health issues;
- Facilitate partnerships with institutions, universities (e.g., University of São Paulo—USP, University of Campinas—UNICAMP, and Universidade Estadual Paulista—UNESP, and non-governmental organizations—NGOs) to increase performance;
- Strengthen the DOMA team.

The DOMA aims to engage in the development of specific programs and projects on the subject of “Information for Action”. It will focus on evaluating exposure to environmental damage and its effects on human health, particularly from chemicals in the air, water and soil [7].

Epidemiological studies enable identify the causes of diseases and the determinants of their distribution, in addition to providing effective knowledge regarding the processes of health and disease [8,9]. Surveillance programs enable the use of Information for Action.

### 1.2. Major Environmental Health Programs and Projects in DOMA

- Health surveillance of populations exposed to atmospheric pollutants;
- Health surveillance of populations exposed to contaminated areas;
- Environmental health surveillance of risks due natural disasters;
- Promote the notification of chemical and environmental emergencies events;
- Promote the investigations of exposed or potentially exposed populations: use of biomarkers (e.g., Billings Dam Project; populations exposed in Baron of Maua Condominium).

## 2. Methodology

This is a descriptive study of the activities of the DOMA, including the notification of environmental events, training and necessary technical definitions of programs for environmental health in the State of Sao Paulo. It analyzes the notifications received between May 2011 and May 2012 and reports the number of notifications received in accordance with the event classification system and analyzes accidents or events related to chemical substances.

The DOMA performs analyses after notifications provided to the Secretary of Health and forwards notifications to regional health departments and municipalities, which establish contacts and investigate the reported events.

The analyses are performed by professionals, including doctors, engineers and biologists. Between May 2011 and May 2012 the DOMA received approximately 300 notifications from the entire State of Sao Paulo. To develop these analyses, the regular activities of the DOMA considered:

*Activities performed by Environmental Health Surveillance*

- Integration with primary health care;
- Family health strategy;
- Situational diagnosis;
- Planning for public health;
- Entrance gateway to the SUS.

*Surveillance actions performed by the SUS planning system*

- Ensuring resources;
- Splits and continuity of actions;
- Strategies for resources negotiation;
- Insertion of integrated actions.

*Social mobilization*

- Risk communication for the people;
- Active participation in councils and participation in the process;
- Decision making;
- Insertion of integrated health actions.

*Parameters and working methodology*

- Environmental Health Criteria/World Health Organization (EHC/WHO) as the criteria for environmental health benchmarks for actions and the environmental health in the State of Sao Paulo [3];
- Environmental epidemiology [10];
- Evaluation of human health risk: Agency for Toxic Substances and Disease Registry/Center of Diseases Control—ATSDR/CDC and recommendation of Ministery of Health from Brazil;
- Databases: Thomes Plus System/RightAnswer, International Agency for Research on Cancer—IARC, Toxicology Data Network—TOXNET, Canadian Cochrane Centre—CCINFO, American Conference of Governmental Industrial Hygienists—ACGIH, and complementary databases [11].

## 3. Results and Discussion

### 3.1. Health Surveillanceof Populations Exposed to Atmospheric Pollutants

The main objectives of the program in the state of Sao Paulo comprise:

- Identify and evaluate the risks and acute and chronic health of populations exposed to pollutants and/or atmospheric pollutants and climate change effects and propose and/or encourage prevention measures, emergency and health promotion of these populations;
- Provide subsidies to public policies that promote prevention and health exposed to pollutants and/or atmospheric pollutants and climate change populations.

Used for analysis risk factors.

#### 3.1.1. Health Risk Factors Associated with Air Pollution in São Paulo

**Figure 1:**
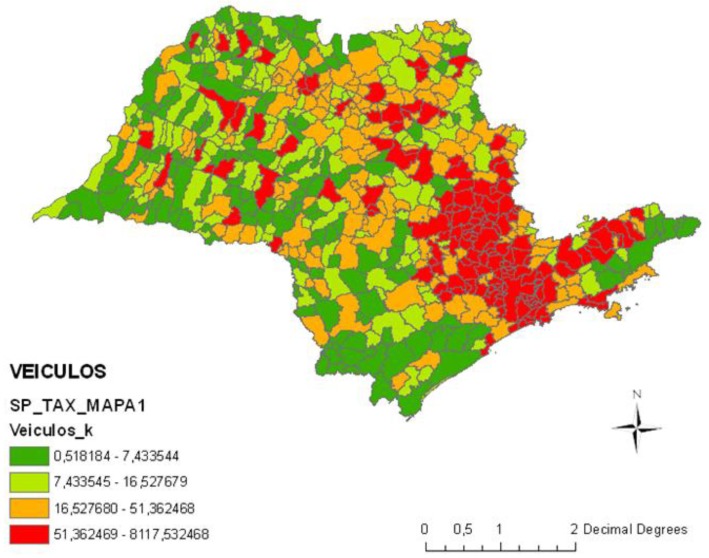
State of Sao Paulo, Brazil—Health risk factors from mobile sources of pollution: vehicles/area.

**Figure 2:**
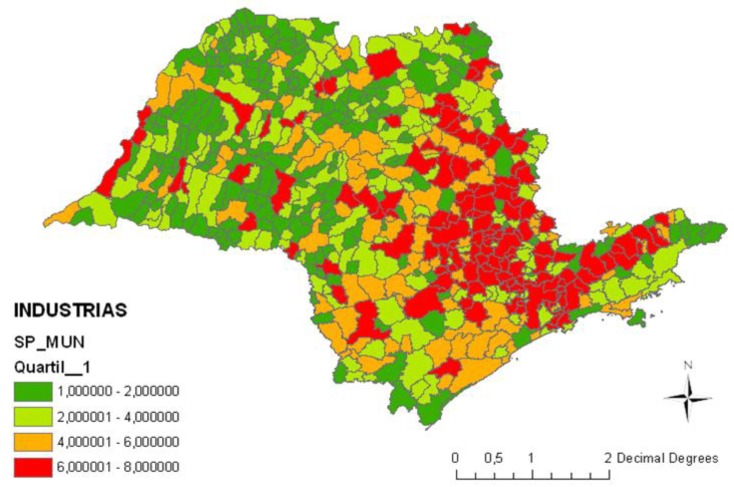
State of Sao Paulo, Brazil—Health risk factors from stationary sources of pollution: industries in the municipality/industries in the State of Sao Paulo.

**Figure 3:**
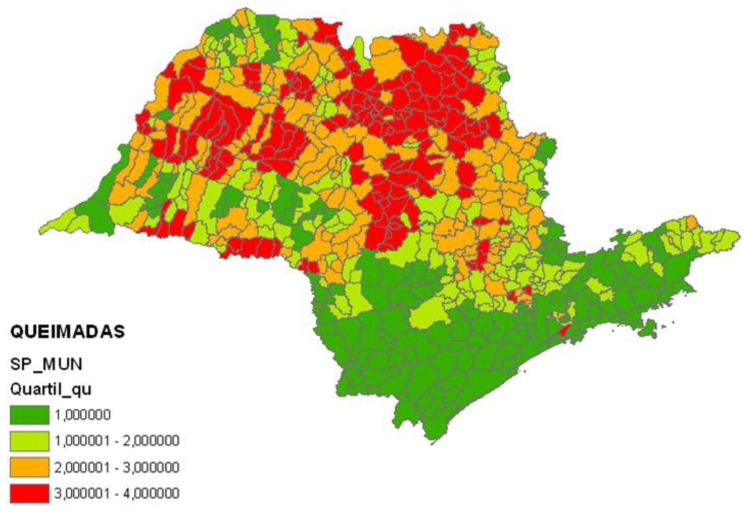
State of Sao Paulo, Brazil—Health risk factors from biomass burning: burning points/area.

**Figure 4:**
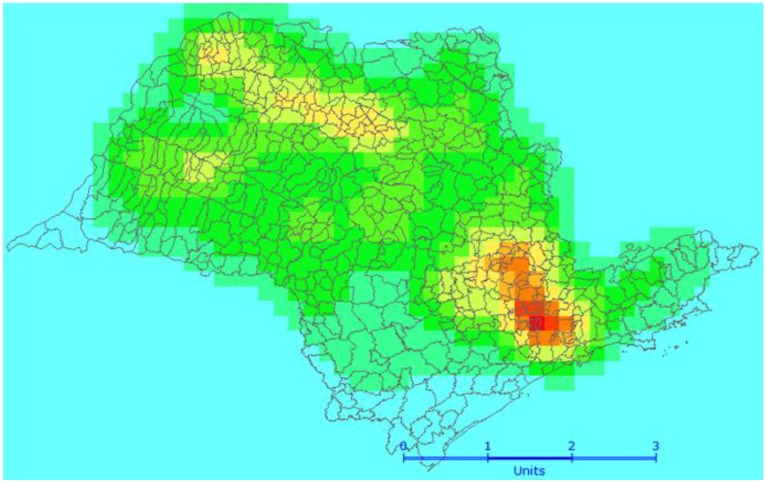
Critical areas (hot spots) for air pollution in Sao Paulo: all sources of air pollution—mobile, stationary and biomass burn.

- Mobili sources: vehicle emissions gases; the index used was vehicles/area (Figure 1);
- Stationary sources: industrial gases and vapors emissions; the index used was industries in the municipality/industries in the State of Sao Paulo (Figure 2);
- Biomass burning: burning of sugarcane; the index used was burning points/area (Figure 3);
- Critical areas for air pollution in the State of Sao Paulo, considering all sources of air pollution (mobile sources + stationary sources + biomass burning) (Figure 4).

#### 3.1.2. Number of Hospitalizations and Deaths

**Figure 5:**
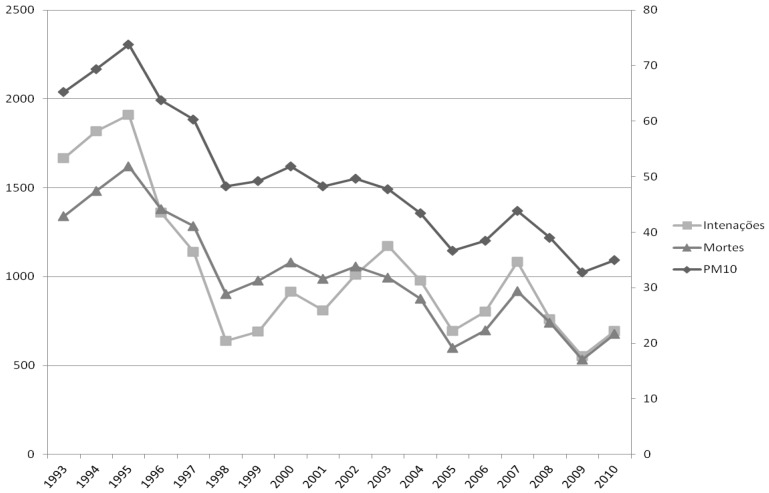
Number of hospitalizations and deaths of children and elderly individuals in Sao Paulo attributable to differences between measurement levels in average air pollution levels. The annual levels of PM_10_ and the standard of 20 μg/m^3^, proposed by the WHO and used as baselines.

In Sao Paulo, a survey of retrospective data from 1993 to 2010, hospitalizations for respiratory diseases and deaths of children and elderly database of the national health system, attributable to the differences between measurement annual levels of particulate matter—PM_10_ and the standard of PM_10_ (20 microns/cubic meter) proposed by the World Health Organization—WHO [12], used as baselines, shows similar curves (Figure 5).

### 3.2. Health Surveillanceof Populations Exposed to Contaminated Areas

**Figure 6:**
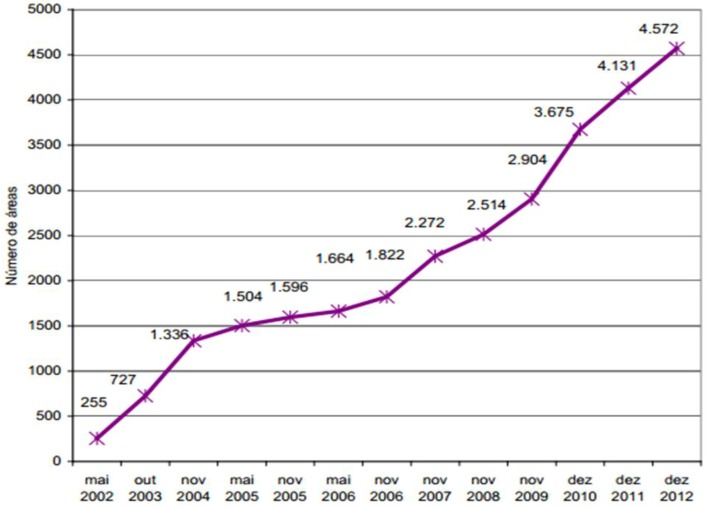
Evolution of the number of contaminated areas, 2002–2013.

**Figure 7:**
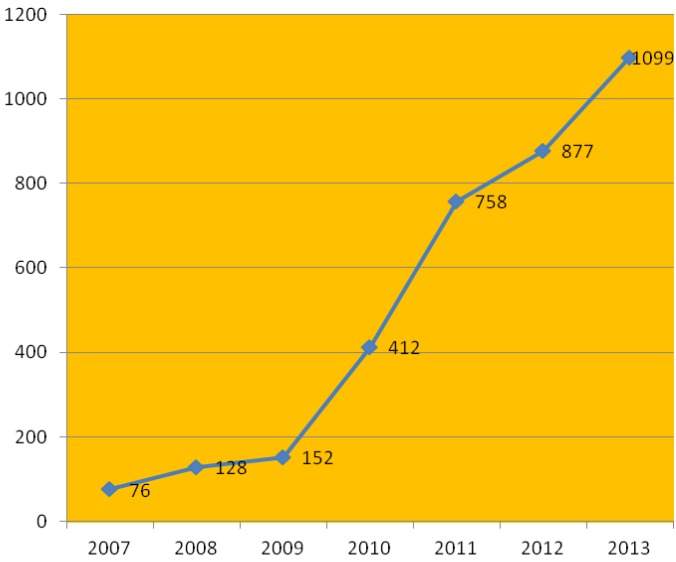
Number of contaminated areas enrolled in the health program, 2007–2013 (Source: Division of Diseases Caused by Environment—DOMA/CVE).

The identification of contaminated areas is performed by an environmental agency. The number of contaminated areas in the State of Sao Paulo has grown substantially. In response, the health agencies have created the corresponding entries in the program. An example is the SISOLO-system of information of contaminated soil, which has been used by health agencies to investigate the population. The identification of contaminated areas is specifically performed by the state environmental agency. The information is provided to the health department, which performs a new analysis that considers the exposed population and the characteristics of the contaminating substances [13]. Notifications have increased primarily (Figure 6) because the environmental agency has conducted further analysis but also as a result of the existence of new industrial areas and unusual destinations of chemicals. The Figure 7 shows the notifications when exist Populations exposed to areas contaminated and area increased too.

Current situation:

- 1099 areas provided to the SISSOLO—system of information of contaminated soil;
- Food conducted by the Secretary of Municipal Health—SMS;
- Gathering environmental and health information;
- Validation of existing information;
- Subsidies for health actions;
- 385 cities with contaminated areas;
- 361 users registered at the SISSOLO (as of 21 June 2013).

Contaminated areas are classified according to the main sources of contamination [14]. 1099 areas identified with population exposed by the health services are classified as Table 1.

**Table 1:** Summary of registered areas, May 2013.

Classification	Number Areas
Area of Disposal of industrial waste	19
Area of urban waste disposal	10
Minning area	1
Deactivated area	89
Industrial areas	124
Pesticides deposits	2
Units of station supplies and services	843
Area contaminated by accident with dangerous goods	11
**TOTAL**	**1099**

**Figure 8:**
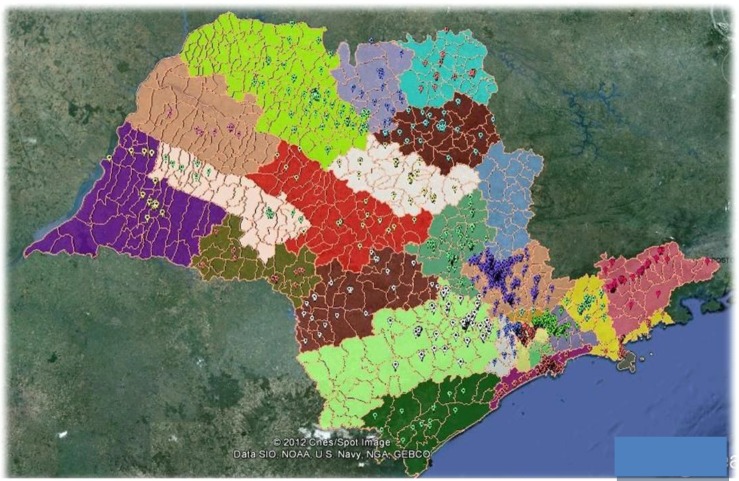
Map of contaminated areas in SP state, 2011.

**Figure 9:**
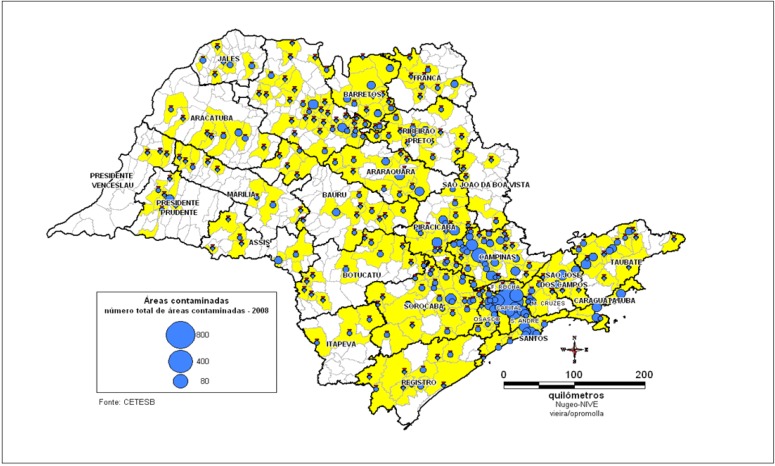
Contaminated areas in SP.

In the next Figure 8 and Figure 9 the distributions of the contaminated areas are shown on maps in the state of Sao Paulo.

The division of health has added technical capacity to the Health Network team.

**Figure 10:**
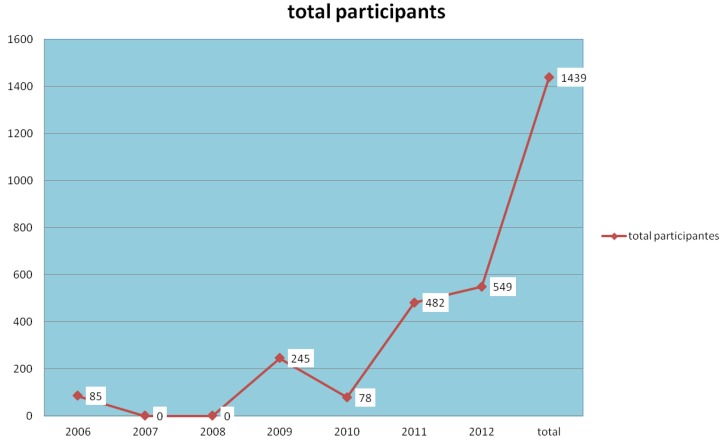
Number of techniques used by the Health Network.

The number of contaminated areas created the need for increased training of technicians to notify and conduct investigations [11]. Table 2 and Figure 10, shows the trainings held, number of people trained (by year).

**Table 2:** Training held to the Health Technicians 2006–2012.

Year	Name of Capacity Building	Number of Trained Individual
2006	Course for Risk Assessment of Human Health Exposure to Hazardous Substance—ATSDR	15
2006	Environmental Epidemiology	40
2006	Course for Risk Assessment of Human Health Exposure to Hazardous Substance—ATSDR	30
2009	Training in Information System of Health Surveillance of Populations	245
2010	Course for Risk Assessment of Human Health Exposure to Hazardous Substance—ATSDR	38
2010	Environmental Epidemiology	40
2011	Workshop VIGISOLO	66
2011	Workshop VIGISOLO—System Information	152
2012	Basic course of Vigisolo—AprilVideo conference—July	280 152

### 3.3. Environmental Event Notification and Chemical Emergencies

**Figure 11:**
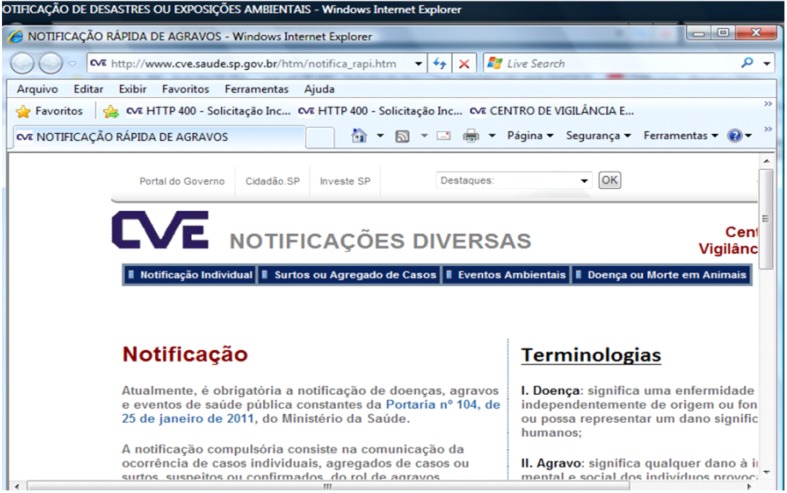
The NOTIFICA ONLINE system.

The notification of environmental events is made by many institutions [15]. The institutions use the system NOTIFICA ONLINE (Figure 11).

**Figure 12:**
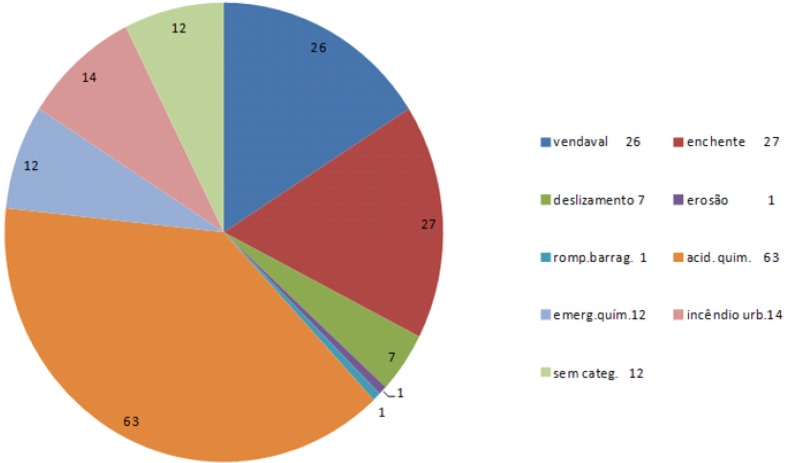
On line notifications sent by CENTRAL-CIEVS to DOMA, Mar 2011–Mar 2012. (39% Chemical accidents; SOURCE: CETESB).

The number of notifications made to Division of Diseases Caused by Environment showed 39% were related to accidents involving chemical substances, followed by natural disasters (flooding), see Figure 12.

Notifications made by the CETESB with impacts on health in the period of Mar 2011–Mar 2012.

There were 57 notifications.

- With impacts on health: 16%
- Without impacts on health: 81%
- Inconclusive: 3%

### 3.4. Other Investigations of Exposed or Potentially Exposed Populations—Use of Biomarkers

**Figure 13:**
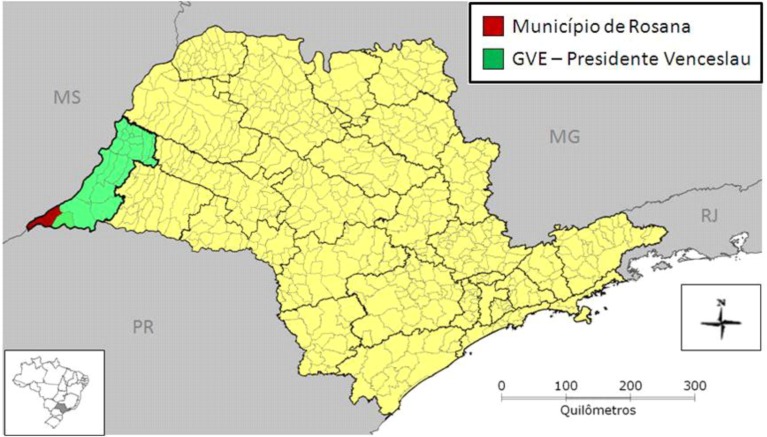
Location of mercury contamination, Municipality of Rosana.

Notes: Pop. 20,646; Area (km^2^) 741.2; Population density (Hab./km^2^) 27.8.

Surveillance epidemiology results (6):

- *1000 individuals* ate contaminated fish *(i.e.*, contaminated with mercury and PCB); cities: Santo André, Diadema, Rio Grande Serra, São Bernardo, SP/Brazil.
- *300 families* were exposed to many substances (*i.e.*, benzene) at a condominium; city: Mauá, SP/Brazil.
- *150 individuals* were exposed to lead; city: S. Sebastião, SP/Brazil.
- *113 cases* = Epidemiological research has identified one hundred and thirty (113) cases of exposure to metallic mercury; of these, six (6) cases were confirmed by laboratory tests such as mercury poisoning (age= 2 to 49).2010; city Rosana, SP/Brazil. The local of the exposition is showed on Figure 13.

### 3.5. Children’s Environmental Health (Sambi)

Proposed health surveillance network system for unified health in the state of SP (SP-SUS). General Objective: Contribute to the promotion, prevention and health surveillance of children (0–14 years) by incorporating the theme of the Children’s Environmental Health Network SUS-SP [16,17]. Three Primary Axes are:

1. PROMOTION AND PREVENTION (e.g., elaborate technical materials, the Stork Network, Network Live, Sec Education, Housing, and Transport).
2. SET SURVEILLANCE (situation diagnosis and surveillance).
3. CARE (train doctors and other professionals from the SUS to investigate and follow up on children with environmental exposure to chemical factors).

**Figure 14:**
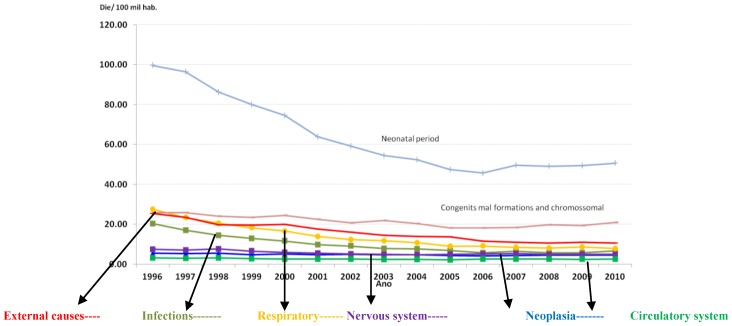
Causes of child mortality in Brazil (1996–2010).

The Infant mortality has major fall, but the congenital malformations continue drawing attention as well as the stability of malignancies in childhood. Figure 14 shows the main causes of infant mortality in Brazil [7].

## 4. Conclusion and Proposal

The analysis of data generated by notifications of events maintained by the Division’s team, which is composed of doctors, engineers and biologists, assured the use of important information; this information aids in the practice of environmental health surveillance at the regional and municipal levels. Based on these analyses, a publication denoting structured guidelines was created. It is entitled “Notebook Surveillance of Epidemiological Environmental Health” [6] and it guides the health services in this area.

Our challenges:

- Contribute to the implementation of the EHS in the State of SP and enable network monitoring consistent with the policies of the SUS.
- Identify and predict the evolution of epidemiological processes via continuous analysis of mortality data on diseases and disorders related to environmental contaminants (*i.e.*, air, water, and soil) at the state level, according to the institutional responsibility of the government.
- Participate and stimulate discussion regarding health institutions and forums considering the characteristics of the state of SP and increase the implementation of measures that directly impact promotion.

The Epidemiological Notifications of Environment Events in São Paulo, Brazil is a good instrument to increase measures that support the identification, action and prevention in the public health arena. However, it is necessary to perform actions together. This necessity is especially true in the arena of laboratory research.
